# Combination chemotherapy of valproic acid (VPA) and gemcitabine regulates STAT3/Bmi1 pathway to differentially potentiate the motility of pancreatic cancer cells

**DOI:** 10.1186/s13578-019-0312-0

**Published:** 2019-06-18

**Authors:** Hehe Li, Zhengle Zhang, Chenggang Gao, Shihong Wu, Qingke Duan, Heshui Wu, Chunyou Wang, Qiang Shen, Tao Yin

**Affiliations:** 1 Department of Pancreatic Surgery, Union Hospital, Tongji Medical College, Huazhong University of Science and Technology, Wuhan, 430022 China; 2 Department of Pancreatic Surgery, Renmin Hospital, Wuhan University, Wuhan, 430060 China; 3 Department of Clinical Cancer Prevention, The University of Texas MD Anderson Cancer Center, Houston, TX 77030 USA

**Keywords:** Pancreatic cancer, Gemcitabine, Valproic acid, Combined chemotherapy, Bmi1

## Abstract

**Background:**

Gemcitabine is the standard first-line chemotherapy regimen for pancreatic cancer. However, its therapeutic value is substantially limited in pancreatic cancer patients due to occurrence of resistance towards gemcitabine. A strategy of combined chemo-regimens is widely employed in clinical settings in attempt to reduce the chance of developing therapeutic resistance. Valproic acid (VPA) has been reported as a promising anticancer drug in various clinical trials and studies. However, the clinical value and potential dose–effect of VPA in combination with gemcitabine for pancreatic cancer treatment are under investigated.

**Results:**

In this study, we determined the synergistic effect of VPA and gemcitabine and found that high-dose VPA significantly and dose-dependently enhanced the sensitivity of pancreatic cancer cells to gemcitabine. Intriguingly, low-dose VPA potentiated the migration and invasion of pancreatic cancer cells that already showed gemcitabine-induced motility. Moreover, low-dose VPA increased the reactive oxygen species (ROS) production, which activated AKT to further stimulate the activation of STAT3, Bmi1 expression and eventually promoted the migration and invasion of pancreatic cancer cells induced by gemcitabine. Whereas high-dose VPA stimulated excessive ROS accumulation that promoted p38 activation, which suppressed the activation of STAT3 and Bmi1.

**Conclusion:**

Pancreatic cancer cells respond differentially towards low- or high-dose of VPA in combination with gemcitabine, and a low VPA further potentiate pancreatic cancer cell to migrate and invade. Our results suggest that STAT3/Bmi1 signaling cascade, which is regulated by ROS-dependent, AKT- or p38-modulated pathways, primarily mediated the sensitivity and motility of pancreatic cancer cells towards combined gemcitabine and VPA regimen. These findings suggest a highly clinically relevant new mechanism of developing resistance against combined chemo-regimens, warranting further mechanistic and translational exploration for VPA in combination with gemcitabine and other chemotherapies.

## Background

Pancreatic cancer is one of the most lethal malignant neoplasms worldwide, with an estimated 5-year survival rate of less than 5% [1]. Intractable drug resistance is accepted as an important causation leading to a low efficacy of chemotherapy in pancreatic cancer [2–4]. It should be noted that current nonsurgical therapeutic options are mostly ineffective, which is largely attributed to the rapid development of drug resistance [2]. Developing effective therapeutic options and sensitizing pancreatic cancer to chemotherapy may be of great importance for improving the treatment efficacy of pancreatic cancer.

Currently, gemcitabine is widely used as a standard first-line regime in pancreatic cancer chemotherapy. Unfortunately, persistent gemcitabine resistance is still the major barrier in the efficacy of pancreatic cancer chemotherapy [3–5]. Recently, a certain concentration of gemcitabine has been indicated to promote the stemness and chemoresistance of pancreatic cancer, which also leads to an increase in migration and invasion [6]. Therefore, understanding the molecular mechanisms leading to chemoresistance and developing effective measures for sensitizing chemotherapy may be of great significance for pancreatic cancer. Recent progress has shown that gemcitabine combined with specific drugs can improve the overall survival of pancreatic cancer patients [7]. Developing combined therapeutics for exerting synergistic effects and reducing drug resistance may be promising strategies for combating pancreatic cancer [8–10].

Histone deacetylases (HDACs) are key components of the epigenetic machinery regulating gene expression that behave as oncogenes in many cancer types [11]. Histone deacetylase inhibitors (HDACIs) exert anticancer activity by inducing cell differentiation and/or apoptosis, as well as by enhancing chemotherapy cytotoxicity [12, 13]. Thus, HDACIs are considered promising anticancer agents and have been approved and used in clinical trials to treat various types of cancer, including pancreatic cancer [14–16].

Valproic acid (VPA) is an inhibitor of histone deacetylase I. It is less toxic by itself and has been tested as an antitumor agent in a clinical trial [17, 18]. Recently, VPA has been extensively studied for its anticancer effect as an adjuvant in combination with a variety of other anticancer agents for many types of cancers, including pancreatic cancer [19, 20]. VPA has been found to sensitize gemcitabine-induced cytotoxicity in gemcitabine-resistant pancreatic cancer cells in an in vitro study [21]. In a clinical phase I/II trial, the combination therapy of VPA and S-1 for patients with pancreatobiliary tract cancers showed a manageable safety profile and preliminary antitumor activity [22]. However, the definitive role of VPA in the chemotherapy of pancreatic cancer has not been clarified until now. It is worth noting that several reports found that VPA induced tumor migration and invasion, which promoted the malignant progression of tumors [23, 24]. Whether VPA induces or inhibits the migratory and invasive capability in pancreatic cancer cells remains unclear and needs to be verified before VPA is used in sensitizing pancreatic cancer chemotherapy.

Previous studies reported that gemcitabine could promote the invasiveness and malignancy of pancreatic cancer, which could be one of the underlying causes of pancreatic cancer chemoresistance [6]. Based on this finding, the synergistic effect of VPA on gemcitabine was detected. We found that high-dose VPA treatment exerted its cytotoxic effect synergized with gemcitabine on pancreatic cancer. Interestingly, low-dose VPA clearly promoted the acquired migration and invasion induced by gemcitabine in pancreatic cancer cells. In addition, our data suggested that activation of the STAT3/Bmi1 signaling cascade by ROS-dependent, AKT- or p38-modulated pathways mediated this process. Our study demonstrated a new phenomenon and mechanism of VPA synergized with gemcitabine in the treatment of pancreatic cancer. VPA should be carefully evaluated before it is used for combating pancreatic cancer.

## Result

### VPA potentiates the motility of pancreatic cancer together with gemcitabine in a concentration dependent manner

**Fig. 1:**
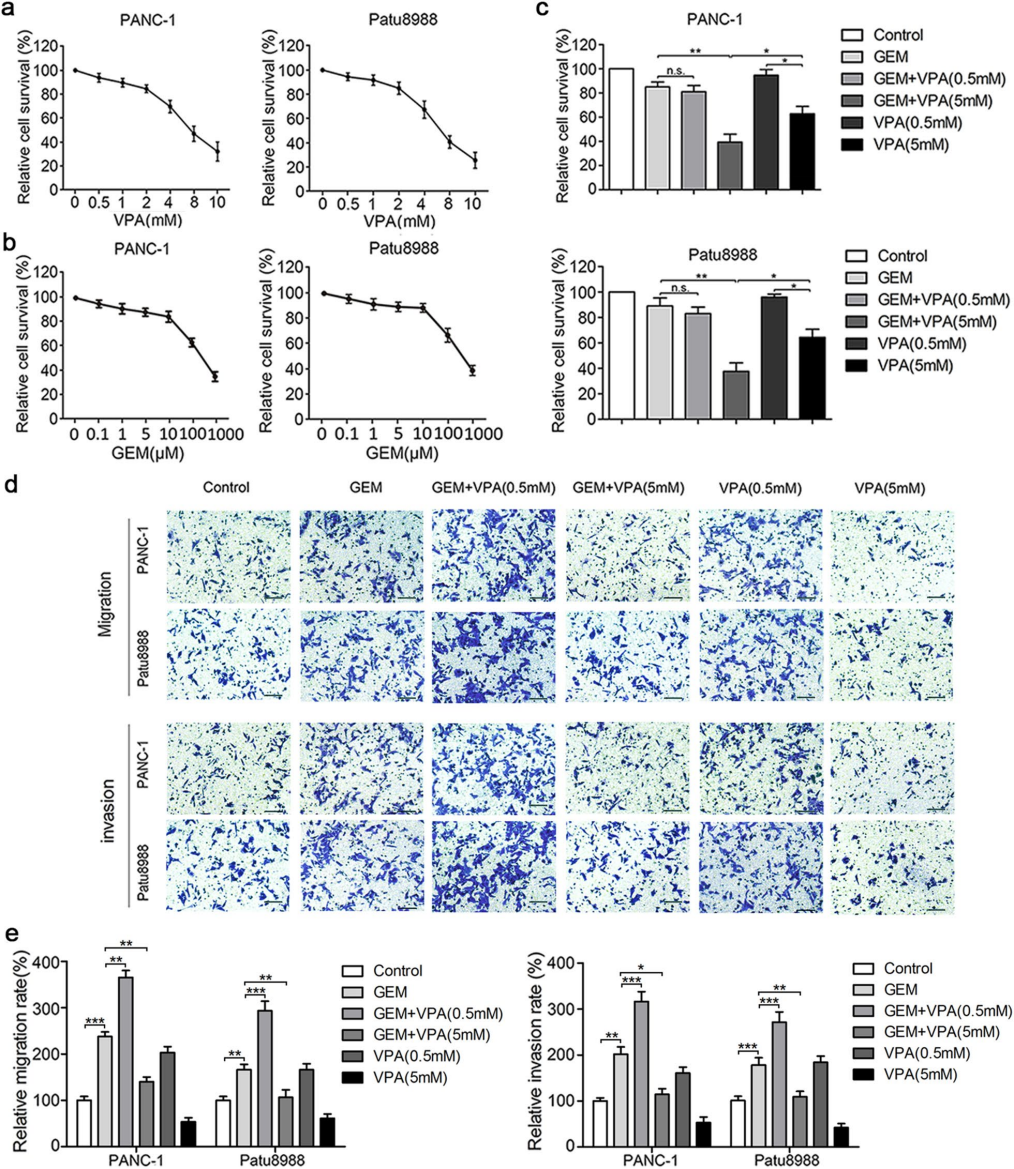
Gemcitabine and VPA affect the cell viability, migration and invasion of pancreatic cancer cells. PANC-1 and Patu8988 cells were treated with 0–10 mM of VPA for 36 h (**a**) or 0–1000 μm of gemcitabine for 24 h (**b**), and the relative survival rate was determined by the MTT assay. **c** After treatment with 0.5 mM or 5 mM VPA for 12 h, both cell lines were treated in combination with 5 μm gemcitabine for 24 h, and cell viability was measured by the MTT assay. **d** The pancreatic cancer cell lines were treated as indicated above, and the migration and invasion abilities were measured by the Transwell migration and invasion assays, respectively. **e** The relative migration/invasion rate were represented motility abilities of pancreatic cancer cells, and it was calculated by counting the migrated/invasive cells in each treatment group relative to the control group. In **a**–**e**, the data are shown from three independent experiments. *n.s.* no significance. *P < 0.05; **P < 0.01; ***P < 0.001 compared with the control

Previous research has reported that gemcitabine could enhance the chemoresistance, migration and invasion of pancreatic cancer cells at relatively low concentrations [6]. Based on this study, the synergetic effects of VPA on gemcitabine were further tested on pancreatic cancer cells in this study. First, the cytotoxicity of gemcitabine and VPA in PANC-1 and Patu8988 cells was detected by MTT assay, and the result showed that VPA treatment exerted its cytotoxic effect in a dose-dependent manner (Fig. 1a, b). Based on our previous and present data, 5 μM of gemcitabine, and 0.5 mM and 5 mM of VPA were chosen for further study. As a result, low-dose gemcitabine (5 µM) showed minimal cytotoxic effects on pancreatic cancer cells, and the combined treatment with low-dose VPA (0.5 mM) showed no significant difference. However, high-dose VPA (5 mM) synergistically enhanced the cytotoxic effect of gemcitabine, which indicated that the concentration of VPA might affect the efficacy of gemcitabine in pancreatic cancer chemotherapy (Fig. 1c).

We further tested the invasion and migration of two pancreatic cancer cell lines cotreated with VPA and gemcitabine. Remarkably, 0.5 mM of VPA collaboratively promoted the invasive and migratory abilities of pancreatic cancer cells induced by gemcitabine (5 µM). However, high-dose VPA (5 mM) significantly attenuated the invasion and migration of pancreatic cancer induced by gemcitabine (Fig. 1d, e). Taken together, our results suggest that VPA could promote the migration and invasion of pancreatic cancer cells induced by gemcitabine in a concentration-dependent manner.

### Low-dose VPA collaboratively promotes gemcitabine-induced Bmi1 expression

**Fig. 2:**
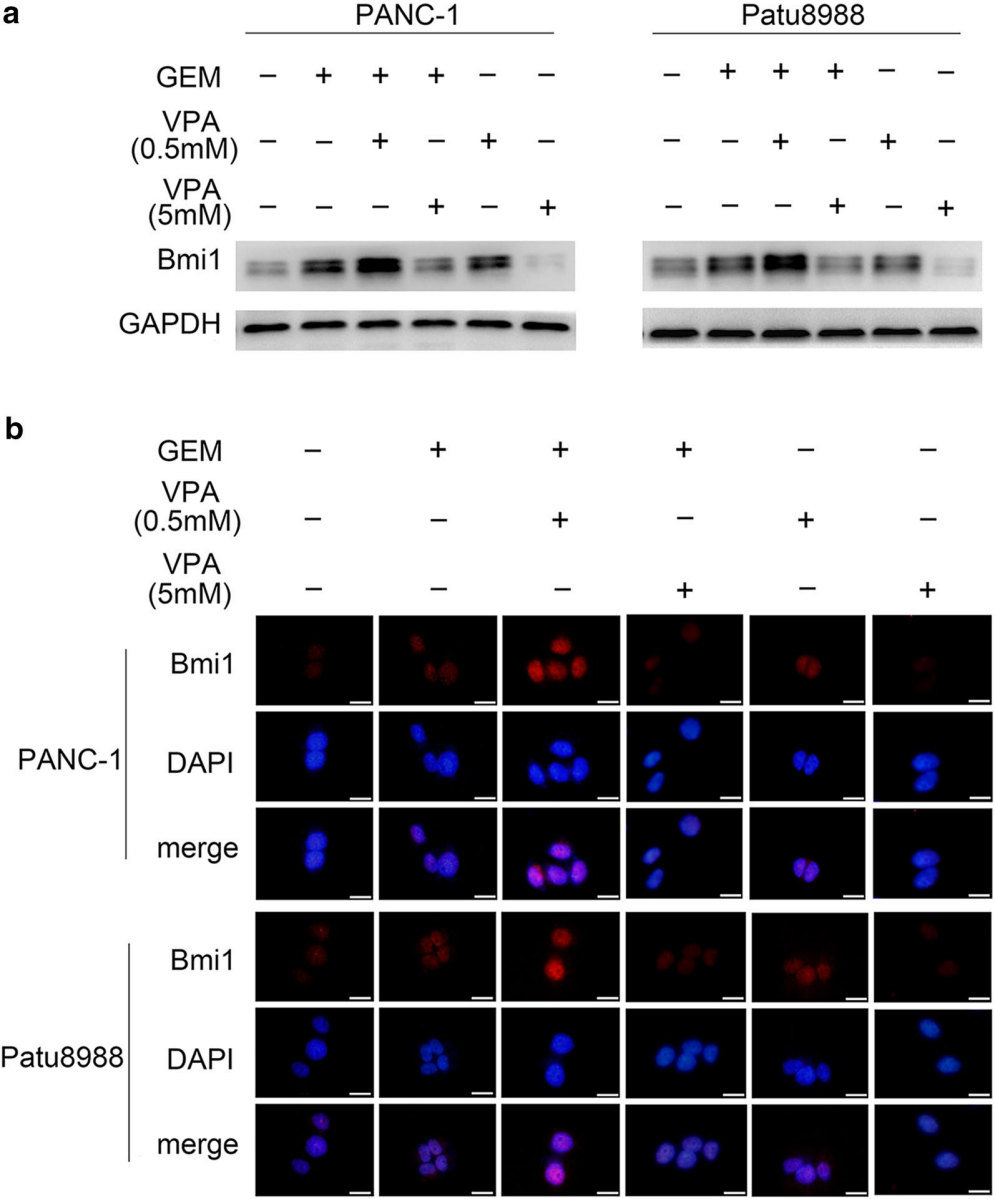
Combination of gemcitabine and VPA regulates Bmi1 expression. PANC-1 and Patu8988 cells were pretreated with 0.5 mM or 5 mM of VPA for 12 h and then cotreated with 5 μm of gemcitabine for 24 h. **a** The protein level of Bmi1 was measured by western blot analysis. **b** The nuclear accumulation of Bmi1 was determined by immunofluorescence. The graphs are representative results of three independently repeated experiments

Bmi1 has been proven to be an important factor in promoting the chemoresistance of pancreatic cancer cells induced by gemcitabine [6, 25]. In this study, PANC-1 and Patu8988 cells were cotreated with gemcitabine and VPA, and the changes in Bmi1 were detected by western blot and immunofluorescence. Interestingly, our results illustrated an increased expression of Bmi1 cotreated with low-dose VPA (0.5 mM) and gemcitabine, whereas Bmi1 decreased after gemcitabine treatment combined with high-dose VPA (5 mM) (Fig. 2a). Immunofluorescence further verified these changes in Bmi1 (Fig. 2b). Taken together, our results suggest that low-dose VPA collaboratively promotes gemcitabine-induced Bmi1 expression, whereas high-dose VPA contradicts Bmi1 expression.

### Low-dose VPA enhances gemcitabine-induced migration and invasion by targeting Bmi1

**Fig. 3:**
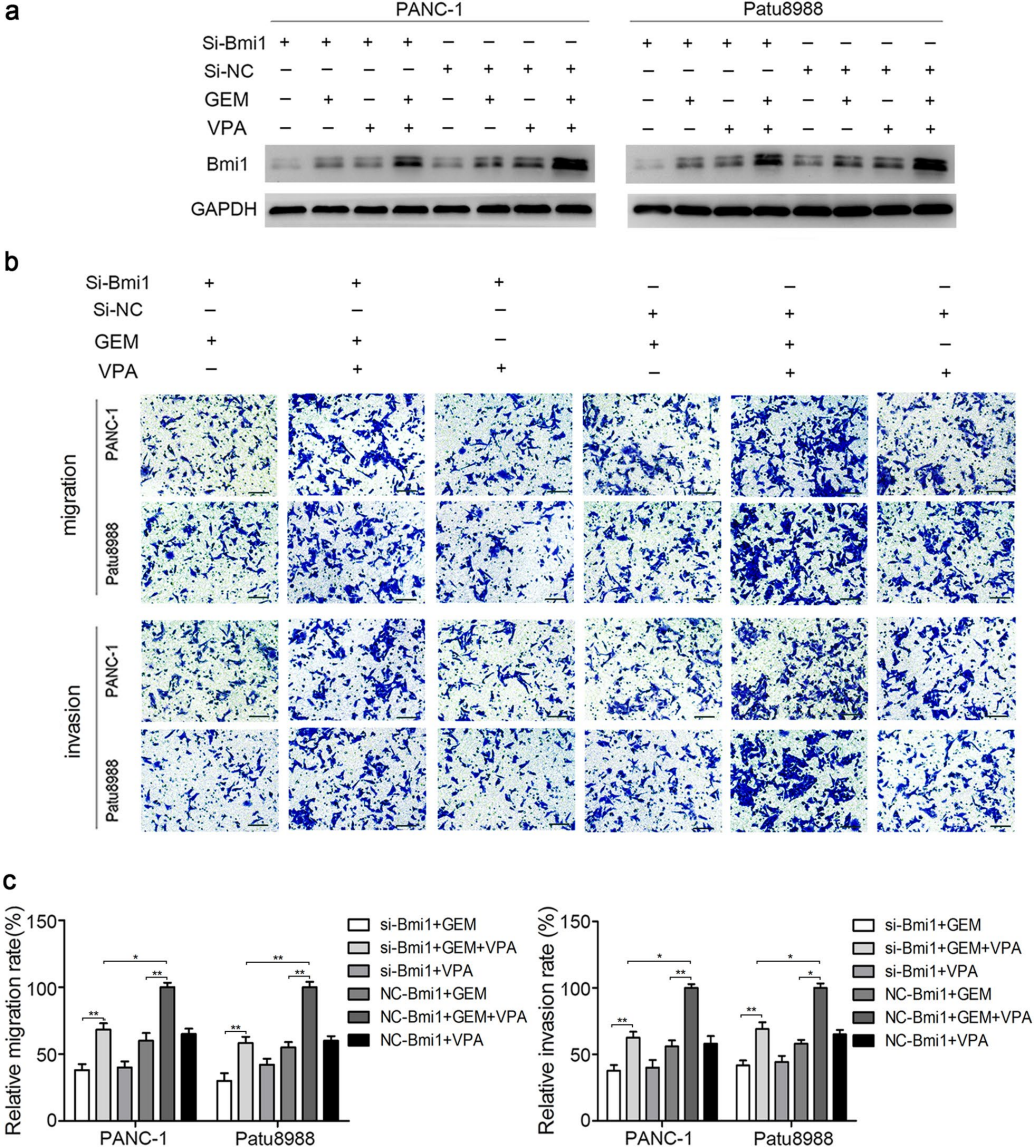
Low-dose VPA enhances gemcitabine-induced migration and invasion by targeting Bmi1. Two pancreatic cancer cells were transfected with Bmi1siRNA and NCsiRNA for 24 h and then treated with 0.5 mM of VPA for 36 h, 5 μm of gemcitabine for 24 h separately and combined. **a** The expression level of Bmi1 was detected by western blot analysis. **b**, **c** The changes in migratory and invasive abilities were evaluated by Transwell migration/invasion assays. The graphs shown are representative results of three independent analyses. *P < 0.05; **P < 0.01; ***P < 0.001 compared with the control

We further detected the role of Bmi1 in the acquired invasion and migration induced by low-dose VPA in combination with gemcitabine. SiRNA was used for silencing Bmi1, and the invasion and migration of pancreatic cancer cells were further investigated. The silencing effect of Bmi1 siRNA was verified by the remarkable reduction of Bmi1 detected by western blot analysis, besides, gemcitabine and VPA alone or combined treatment partly recover the Bmi1 reduction (Fig. 3a). After Bmi1 was inhibited, Transwell assays showed that the migration and invasion of pancreatic cancer cells were reduced by gemcitabine and low-dose VPA separately and combined therapy. The results indicated that Bmi1 contributed to the acquired migration and invasion induced by gemcitabine in combination with low-dose VPA treatment (Fig. 3b, c).

### STAT3 is involved in mediating the gemcitabine/low-dose VPA-induced migration and invasion of pancreatic cancer cells

**Fig. 4:**
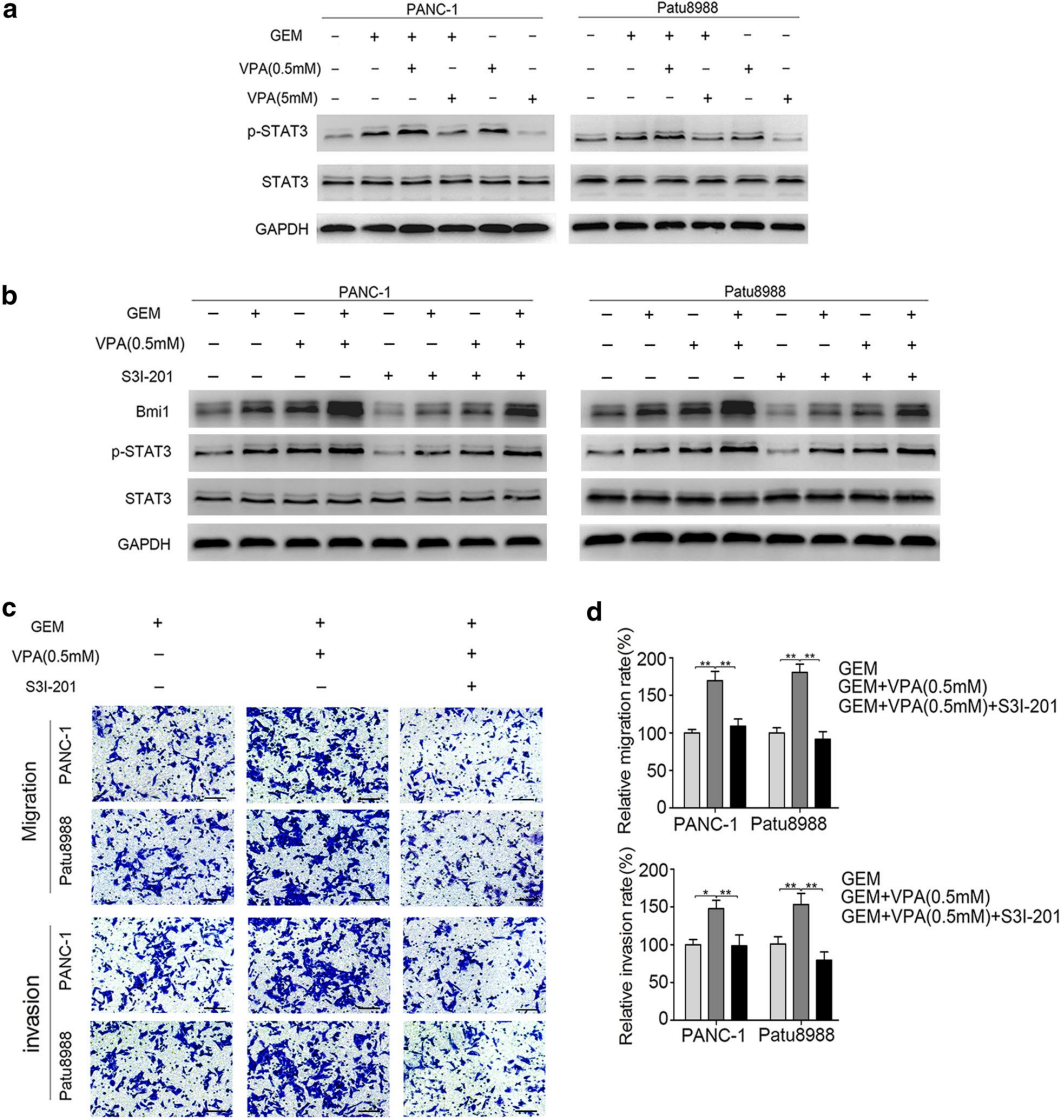
STAT3 is involved in mediating the gemcitabine/low-dose VPA-induced migration and invasion of pancreatic cancer cells. **a** PANC-1 and Patu8988 cells were cotreated with 5 μm of gemcitabine for 24 h and various doses of VPA for 36 h, and the expression levels of p-STAT3 and STAT3 were detected by western blot analysis. Two pancreatic cancer cells were pretreated with 100 μm of S3I-201 for 4 h and then treated with 0.5 mM of VPA for 12 h; followed by cotreatment with gemcitabine for 24 h. **b** The changes in Bmi1, p-STAT3, and STAT3 were measured by western blot analysis. **c**, **d** The migration and invasion were detected by Transwell assays after the above treatment. *P < 0.05; **P < 0.01; ***P < 0.001 compared with the control

The STAT3 signaling pathway plays an important role in the progression of chemoresistance among pancreatic cancer cells [26, 27]. We further detected the role of STAT3 in the acquired migration and invasion of pancreatic cancer induced by gemcitabine and VPA. Two pancreatic cancer cell lines were treated with different concentrations of VPA with or without gemcitabine for the indicated time, and the expression of STAT3 was observed. In this study, low concentrations of gemcitabine promoted STAT3 activation, and low-dose VPA (0.5 mM) further enhanced this effect. However, high-dose VPA (5 mM) dramatically suppressed STAT3 activation (Fig. 4a). In addition, when pretreated with S3I-201, a selective inhibitor of STAT3, and the collaborative inductive effect of Bmi1 was also clearly weakened (Fig. 4b). Moreover, both cell lines cotreated with gemcitabine and low-dose VPA exhibited significantly reduced migration and invasion after STAT3 inhibition (Fig. 4c, d). These observations indicated that VPA might modulate the acquired invasion and migration induced by gemcitabine mainly through regulating STAT3 activation and Bmi1 expression. Taken together, our results suggest that STAT3 plays an important role in the acquired migration and invasion induced by gemcitabine and low-dose VPA cotreatment.

### ROS accumulation modulates the STAT3 activation, migration and invasion induced by gemcitabine and VPA

**Fig. 5:**
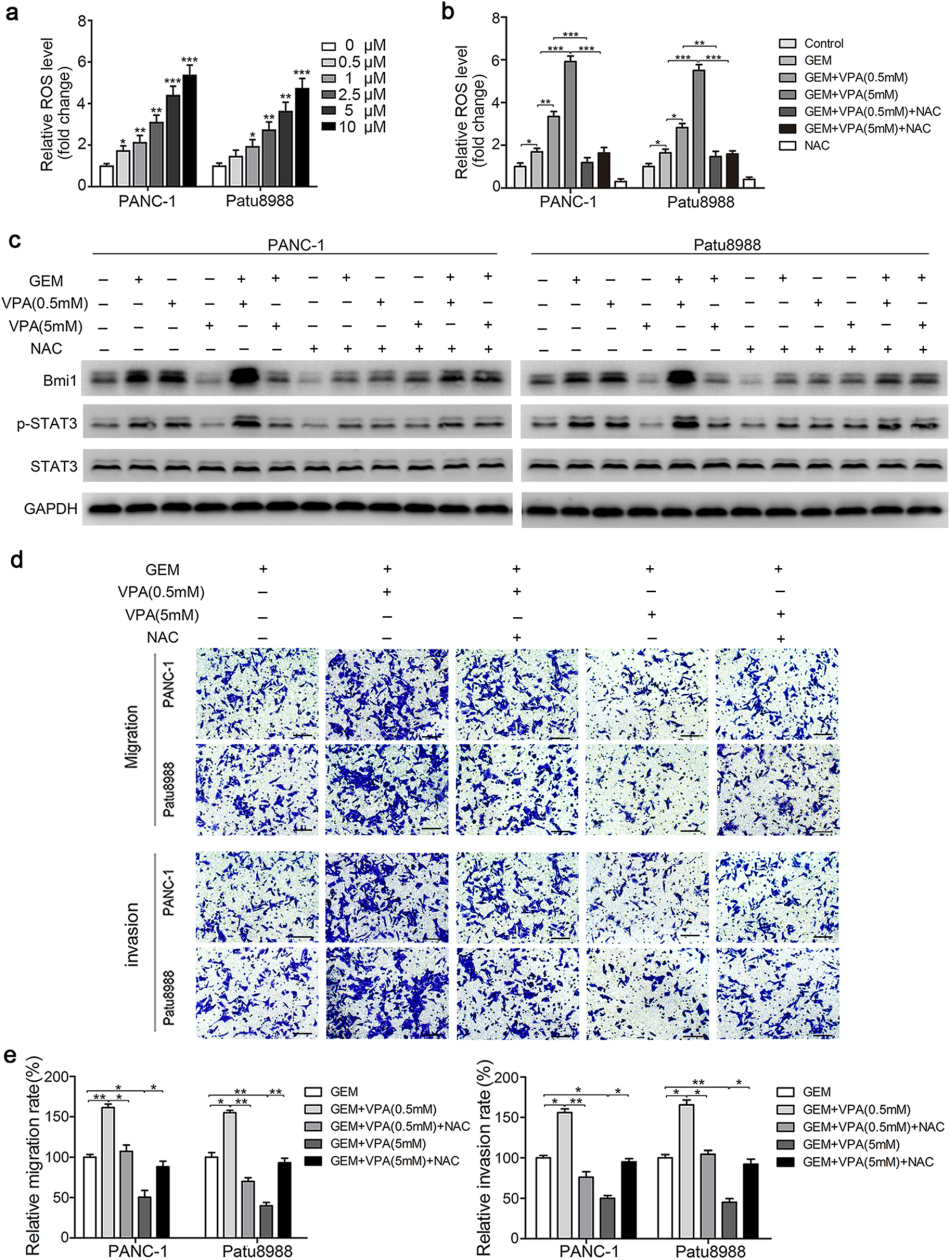
ROS accumulation modulates the STAT3 activation, migration and invasion induced by gemcitabine and VPA. **a** After various doses of VPA treatment for 36 h, the relative intracellular ROS level was examined using a DCF-DA probe with FCM. **b** After pretreatment with 10 mM of NAC for 2 h and then treatment with VPA (0.5 mM or 5 mM) for 12 h in two pancreatic cancer cell lines, cotreatment with gemcitabine was followed for 24 h. Then, the changes in intracellular ROS levels were detected by FCM. **c** After pretreatment with 10 mM of NAC for 2 h and then treatment with VPA (0.5 mM or 5 mM) for 12 h separately and combined with 5 μm gemcitabine for another 24 h, the changes in the expression levels of Bmi1, p-STAT3 and STAT3 were detected by western blot analysis. **d**, **e** The migration and invasion abilities were detected by Transwell migration/invasion assays after the above treatment. *P < 0.05; **P < 0.01; ***P < 0.001 compared with the control. The graphs show the results of three independent experiments

It has been reported that ROS are involved in the chemoresistance and invasion of cancer cells [28–30]. We further elucidated the role of ROS in the enhanced invasion and migration of pancreatic cancer cells. Flow cytometry was used to detect changes in ROS in pancreatic cancer cells. The results showed that VPA treatment increased ROS in a dose-dependent manner (Fig. 5a). VPA promoted ROS production in combination with gemcitabine, which can be attenuated by the presence of antioxidant NAC (Fig. 5b). Furthermore, western blot analysis revealed that both the STAT3 activation and Bmi1 expression induced by gemcitabine and low-dose VPA (0.5 mM) could be inhibited after ROS were scavenged by NAC. Interestingly, NAC treatment partially rescued the reduced Bmi1 expression and p-STAT3 induced by gemcitabine and high-dose VPA (5 mM) (Fig. 5c). In addition, the enhanced invasion and migration induced by low-dose VPA and gemcitabine was significantly attenuated after ROS were scavenged by NAC (Fig. 5d, e). Taken together, our results suggest that ROS accumulation modulates the STAT3 activation, migration and invasion induced by gemcitabine and VPA.

### AKT mediates ROS-induced STAT3 activation induced by gemcitabine and low-dose VPA

**Fig. 6:**
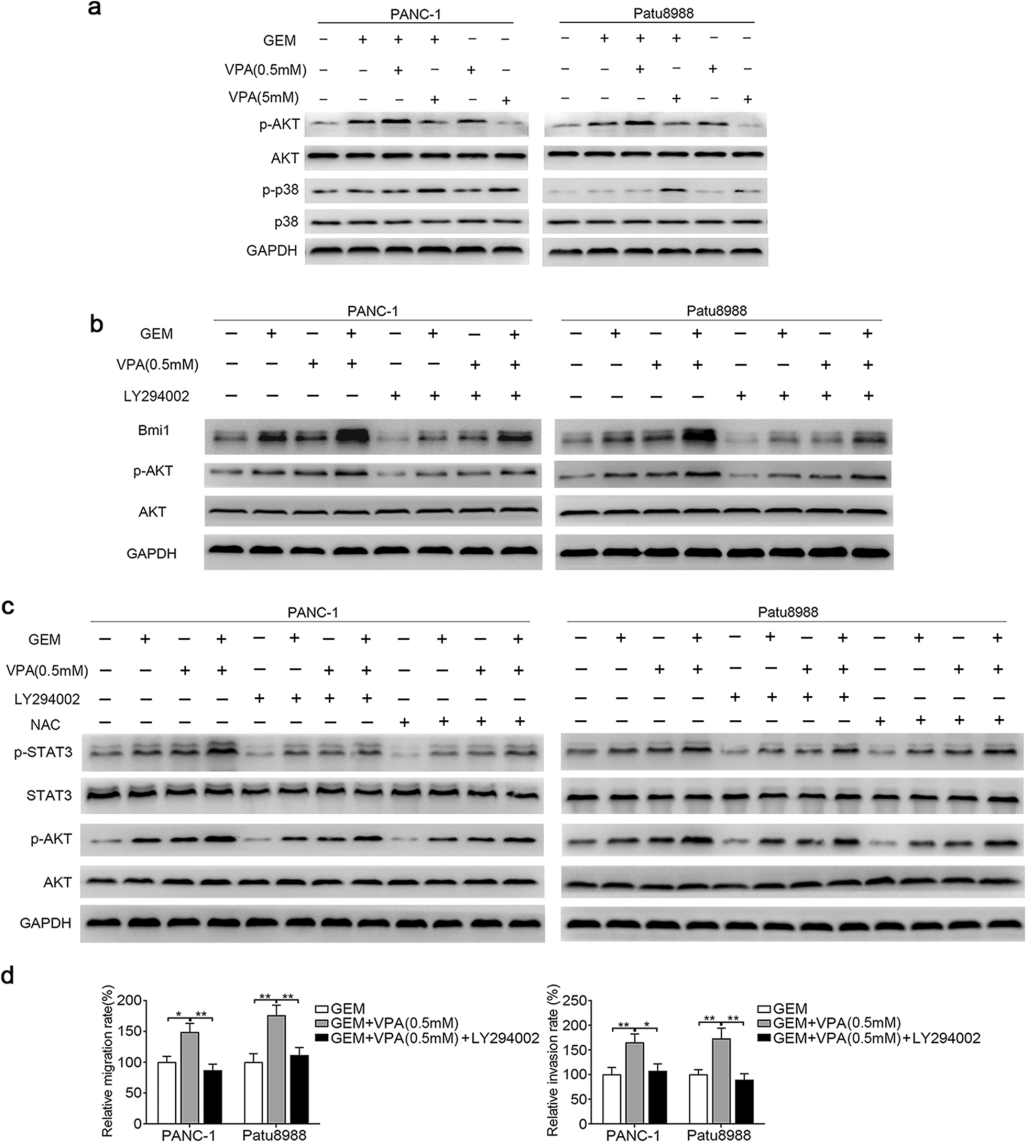
AKT mediates ROS-induced STAT3 activation induced by gemcitabine and low-dose VPA. **a** Two pancreatic cancer cell lines were treated with VPA (0.5 mM or 5 mM) for 12 h, followed by cotreatment with gemcitabine for 24 h. The expression levels of p-AKT, AKT, p-p38, and p38 were determined by western blot analysis. **b** PANC-1 and Patu8988 cells were pretreated with 20 μm of LY294002 for 2 h, followed by gemcitabine and 0.5 mM of VPA treatment separately and combined; the expression of Bmi1, p-AKT and AKT was detected by western blot analysis. **c** After pretreatment with 10 mM of NAC or 20 μm of LY294002 for 2 h, followed by gemcitabine and 0.5 mM of VPA treatment separately and combined, the changes in p-STAT3, STAT3, p-AKT and AKT were examined by western blot analysis. **d** Two pancreatic cancer cell lines were pretreated with 20 μm of LY294002, followed by gemcitabine and 0.5 mM of VPA cotreatment. The migratory and invasive abilities were examined by Transwell migration/invasion assays. *P < 0.05; **P < 0.01; ***P < 0.001 compared with the control

As a key mediator of many intracellular biological processes, AKT was also associated with migration and invasion of cancer cells [31]. We further investigated the role of AKT in the STAT3 activation induced by gemcitabine and VPA cotreatment, and these findings showed that low-dose VPA (0.5 mM) promoted AKT activation, while VPA at high concentration (5 mM) inhibited AKT activation (Fig. 6a). Moreover, the presence of gemcitabine can reinforce this effect in combination with VPA. We pretreated the two pancreatic cancer cell lines with an AKT inhibitor (LY294002, 20 μm) before gemcitabine and low-dose VPA (0.5 mM) separately or combined treatment. Interestingly, a weakened upregulation of Bmi1 and STAT3 activation was observed after AKT was inhibited (Fig. 6b, c). Consistently, the migration and invasion of both pancreatic cancer cell lines were weakened after AKT was inhibited (Fig. 6d). Interestingly, NAC also downregulated p-AKT expression, which was positively correlated with p-STAT3 (Fig. 6c). Taken together, our results suggest that ROS stimulates the activation of AKT and STAT3, and the migration and invasion of pancreatic cancer cells induced by gemcitabine and low-dose VPA.

### Excessive ROS promotes p38 activation and suppresses the migration and invasion of pancreatic cancer cells

**Fig. 7:**
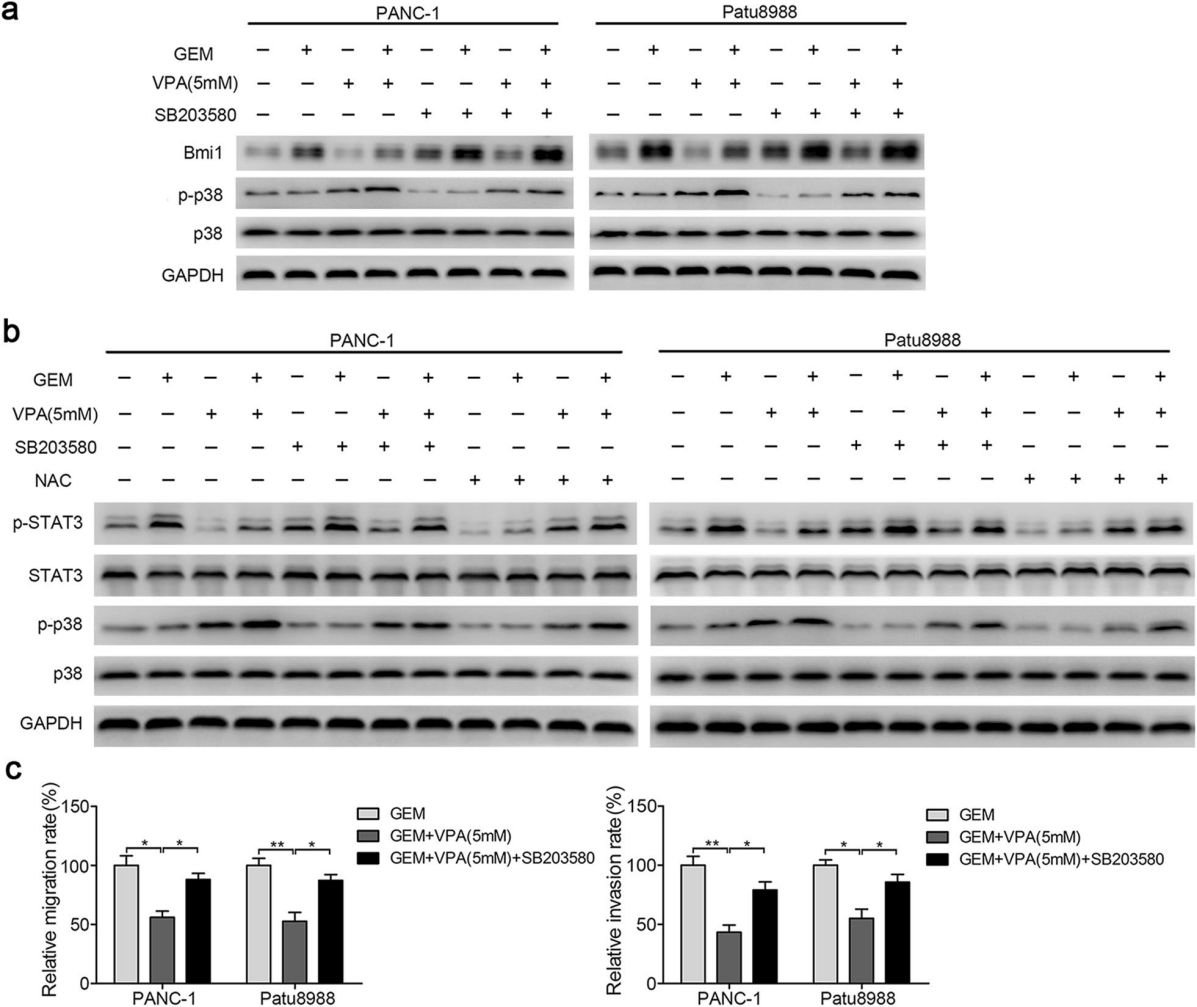
Excessive ROS promotes p38 activation and suppresses the migration and invasion of pancreatic cancer cells. **a** PANC-1 and Patu8988 cells were pretreated with 10 μm of SB203580 for 2 h and then treated with 5 mM of VPA for 12 h, followed by cotreatment with gemcitabine for 24 h. The expression of Bmi1, p-p38 and p38 was detected by western blot analysis. **b** After pretreatment with 10 mM of NAC or 10 μm of SB203580 for 2 h, western blotting was performed to examine the changes in p-STAT3, STAT3, p-p38 and p38. **c** The migration and invasion were examined by Transwell assays after 10 μm SB203580 pretreatment for 2 h, followed by gemcitabine and 5 mM VPA cotreatment. *P < 0.05; **P < 0.01; ***P < 0.001 compared with the control

We further elucidated the mechanism leading to the decreased migration and invasion of pancreatic cancer cells by gemcitabine and high-dose VPA. Produced by oncogenes in tumorigenesis, p38 is a special sensor of ROS [32]. In our study, high-dose VPA (5 mM) significantly increased the p-p38 levels in pancreatic cancer cells, as detected by western blot (Fig. 6a). Accordingly, high-dose VPA (5 mM) decreased the gemcitabine-induced Bmi1 expression (Fig. 7a).

Both pancreatic cancer cell lines were pretreated with SB203580, a p38 inhibitor, before treatment with gemcitabine and high-dose VPA (5 mM). P-p38 inhibition partially rescued the reduced Bmi1 expression and partly reestablished migration and invasion in both cell lines tested (Fig. 7a, c). Moreover, the inhibition of p-STAT3 by high-dose VPA (5 mM) was effectively restored when p38 was inhibited (Fig. 7b). In addition, NAC could inhibit the activation of p38 and partially rescue STAT3 activation (Figs. 5d, 7b). Taken together, our results suggest that excessive ROS promote the activation of p38 and the suppression of STAT3 activation induced by gemcitabine and high-dose VPA cotreatment.

## Discussion

Intractable drug resistance is one of the most important causes of poor prognosis in pancreatic cancer patients [2, 3, 7]. The combination of multiple drugs with synergistic effects to reduce resistance may be a promising strategy for combating pancreatic cancer [8–10]. VPA, an inhibitor of histone deacetylase I, has shown promising anticancer effects, especially as an adjuvant in combination with other anticancer agents [19, 21]. Our previous study verified that low-dose gemcitabine could promote the malignancies of pancreatic cancer [6], which may be one of the underlying mechanisms of pancreatic cancer drug resistance. Based on this finding, we further detected the synergistic effect of VPA and gemcitabine on pancreatic cancer chemotherapy. Unexpectedly, we found that VPA treatment exerted its cytotoxic effect in a dose-dependent manner. High-dose VPA significantly increased the sensitivity of pancreatic cancer cells to gemcitabine. However, low-dose VPA, which showed little cytotoxicity on pancreatic cancer cells, clearly promoted the acquired migration and invasion induced by low-dose gemcitabine.

One of the important findings of our study is that the optimal application dose should be given more attention before VPA can be used in clinic to treat pancreatic cancer. We did prove that high-dose VPA sensitized the cytotoxicity of low-dose gemcitabine on pancreatic cancer and inhibit the malignant behaviors of migration and invasion. However, such synergistic effects of VPA are limited for clinical use because this concentration (5 mM) greatly exceeded the upper limit of the antiepileptic range of 0.9 mM [33]. The clinical trials failed to increase the dose of VPA safely because of some neurological side effects, such as confusion and disorientation, were dose-related in patients who were exposed to high VPA serum concentrations [34–36]. More importantly, low-dose VPA (0.5 mM in this study, range of clinically therapeutic dose of VPA is 0.35–0.7 mM [37] was proved to promote the migration and invasion of pancreatic cancer in combination with gemcitabine in our study. Hence, it is valuable to further decipher the underlying efficacy of different concentrations and the combined regimen of VPA before it was used in treating pancreatic cancer.

One of our findings is that Bmi1 is involved in the effect of VPA combined with gemcitabine. Bmi1 was reported to be an oncoprotein, which has crucial relevance to epigenetic regulation and chemotherapy resistance [38–41]. Previous studies have found that Bmi1 plays a promoting role in gemcitabine-induced chemoresistance and migration in pancreatic cancer cells [6]. Our study showed that low-dose VPA significantly enhanced gemcitabine-induced Bmi1 expression, whereas high-dose VPA decreased this change. Knocking down Bmi1 in pancreatic cancer cells suppressed the migration and invasion induced by VPA and gemcitabine cotreatment. These results emphasize the importance of Bmi1 in pancreatic cancer chemoresistance. Developing strategies for targeting Bmi1 may represent promising ways to promote the effects of chemotherapy on pancreatic cancer.

As an oncogene, STAT3 is constitutively activated, which largely leads to chemoresistance and promotes migration and invasion in a variety of cancer cells [42–45]. Gemcitabine could promote the binding of phosphorylated STAT3 to the Bmi1 promoter, which further enhances the stemness and migration of cancer cells [6]. The inhibition of STAT3 signaling may be a promising strategy for attenuating chemoresistance in pancreatic cancer [46]. Despite previous reports claiming that VPA acts as a selective STAT3 inhibitor and can inhibit STAT3 phosphorylation [47], we found more complex interactions between VPA and STAT3. VPA at a high dose exhibited an inhibitory role on STAT3 activation. However, low-dose VPA can even stimulate STAT3 activation, which was consistent with the changes in Bmi1. In addition, the enhanced migration and invasion induced by gemcitabine and VPA co-treatment were suppressed after STAT3 inhibition, which further verified that STAT3 signaling participates in mediating Bmi1 expression and the biological effect induced by VPA and gemcitabine treatment on pancreatic cancer cells induced by induced by VPA and gemcitabine treatment on pancreatic cancer cells.

It has been reported that antitumor drugs could trigger ROS production to exert their cytotoxic ability [48, 49]. Gemcitabine has also been proven to activate ROS to enhance the anticancer effect [50]. In our study, high levels of ROS induced by high-dose VPA promoted the cytotoxic effect of gemcitabine, which indicated that inducing oxidative stress through triggering high ROS may provide promising measures for sensitizing gemcitabine. Recently, there has been an enhanced realization for the role of ROS, which modulate various cellular signaling pathways involved in the proliferation and invasion of cancer [29]. Low-dose gemcitabine has been proven to promote pancreatic cancer cell invasiveness through a reactive oxygen species-dependent mechanism [6, 51]. Our results showed that VPA enhanced ROS accumulation in a dose-dependent manner. Low levels of ROS induced by low-dose VPA reinforced the invasion and migration of pancreatic cancer cells triggered by gemcitabine. Low ROS-mediated signaling pathways may be potential targets for sensitizing pancreatic cancer chemotherapy.

**Fig. 8:**
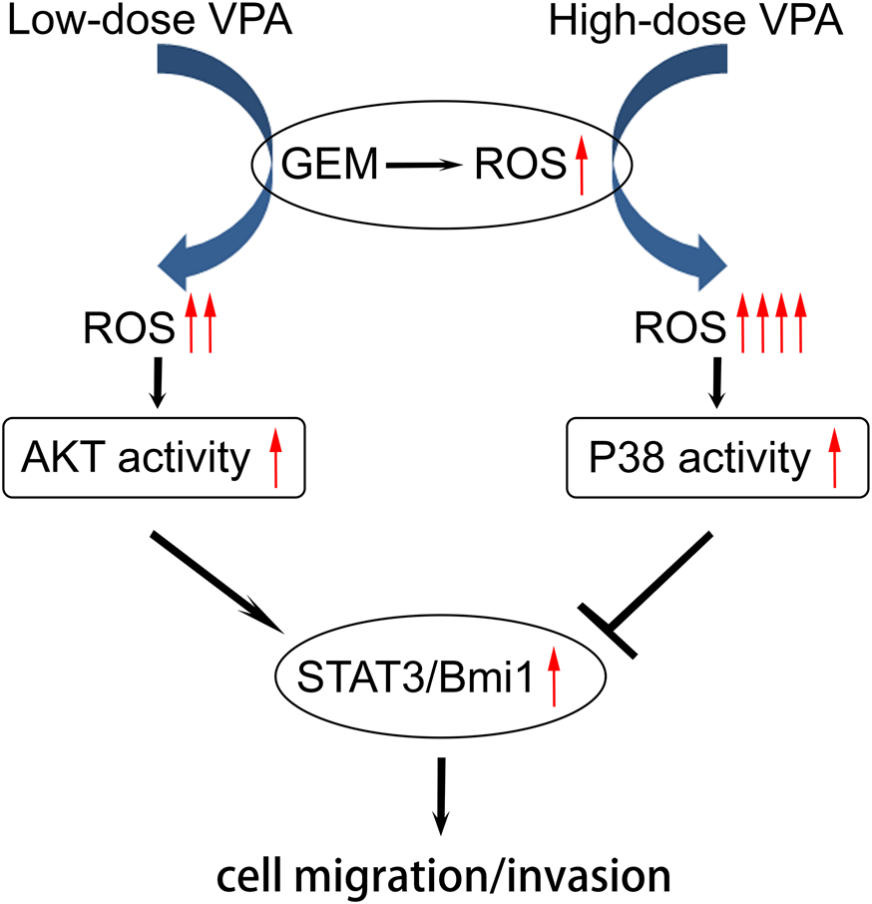
A schematic diagram illustrate the potential mechanisms of VPA and gemcitabine on pancreatic cancer cells

Compelling evidence highlights that both AKT and p38 are crucial and essential signal molecules by which ROS exert their biological impact on cancers [52–54]. In this study, we demonstrated that low-dose VPA triggers AKT activation and promotes the migration and invasion of pancreatic cancer cells. High-dose VPA activates p38 to suppress gemcitabine-induced migration and invasion. Moreover, different levels of ROS activate AKT and p38 differentially in combination with gemcitabine and VPA. Published data have reported that AKT and p38 are all involved in ROS-sensitive pathways [29]. We show that the level of ROS is a fate-determining factor for activating AKT or p38, and distinct biological effects are manifested leading to differential outcomes of cell motility and tumor growth. Furthermore, p38 and AKT may display antagonistic roles after treatment with gemcitabine in combination with different concentrations of VPA. We demonstrated that low levels of ROS-mediated AKT activation [55] and excessive ROS accumulation promote p38 activation. AKT or p38 may act as ROS-dependent regulators to target STAT3 and Bmi1 (Fig. 8). Moreover, the STAT3/Bmi1 axis may be important downstream effectors determining the biological reactions of pancreatic cancer cells during chemotherapy. Our study provides potential targets for sensitizing pancreatic cancer chemotherapy.

One insufficiency of this study is the lack of in vivo validation. The main obstacles to animal experiments are the challenge of ensuring the consistency of differential concentrations of VPA and the difficulty of determining the dose–effect relationship of VPA in vivo. Another consideration is the potential adverse effects using high-dose VPA, which hindered the in vivo validation. Further exploration of this aspect is still under investigation. Nevertheless, our current study strongly suggests that the dose–effect relationship of VPA should be carefully evaluated before application in chemosensitizing and treating pancreatic cancer.

## Conclusions

This study provides evidence that pancreatic cancer cells respond differentially towards low- or high-dose VPA in combination with gemcitabine, and a low VPA further potentiate pancreatic cancer cell to migrate and invade. Our results suggest that STAT3/Bmi1 signaling cascade, which is regulated by ROS-dependent, AKT- or p38-modulated pathways, primarily mediated the sensitivity and motility of pancreatic cancer cells towards combined gemcitabine and VPA regimen. Our findings suggest a highly clinically relevant new mechanism of developing resistance against combined chemo-regimens, warranting further mechanistic and translational exploration for VPA in combination with gemcitabine and other chemotherapies.

## Materials and methods

### Cell culture

The human pancreatic cancer cell line PANC-1 originated from ATCC and was cultured in RPMI-1640 medium at 37 °C in a 5% CO_2_ incubator. The Patu8988 cell line was obtained from Keygen (Keygen Biotech, China) and was cultured in DMEM at 37 °C in a 5% CO_2_ incubator. Both cell culture media were supplemented with 10% fetal bovine serum (Gibco, Grand Island, NY, USA) and a 1% penicillin/streptomycin mixture.

### MTT assay

After digestion and suspension, cells were planted into 96-well plates at a density of 0.6 × 10^4^ cells/well and treated with increasing concentrations of gemcitabine (Selleck.cn, Shanghai, China) (0–1000 μm) for 12 h or VPA (Sigma-Aldrich, St. Louis, MO, USA) (0–10 mM) for 36 h. Each group had five replicated wells. Then, every well was supplemented with 20 μL of MTT (Sigma-Aldrich) (5 mg/mL) reagent and incubated at 37 °C for 4 h in the dark. The medium was replaced with 150 μL of DMSO (Sigma-Aldrich) and incubated in a shaker for 15–20 min. Finally, the absorbance of each well was measured at an excitation wavelength of 490 nm using a microplate reader.

### Transwell migration assay

A migration model of pancreatic cancer cells was constructed using 8 μM Transwell chambers (Costar, Corning, Cambridge, MA, USA). A total of 5 × 10^4^ cells in 200 μL of culture medium plus 0.1% FBS were planted into the upper chamber, and 700 μL of medium containing 30% FBS was added to the lower chamber. After incubation for 24 h, cells were fixed with 4% paraformaldehyde and stained with 0.5% crystal violet. Cells on the underside of the chamber membrane were observed and counted in 5 randomly chosen horizons under an inverted microscope to obtain the average value.

### Transwell invasion assay

A migration model of pancreatic cancer cells was constructed using 8 μM Transwell chambers (Costar) coated with ECM gel (Sigma-Aldrich). 5 × 10^4^ cells in 200 μL of culture medium plus 0.1% FBS were planted into the upper chamber, and 700 μL of medium containing 30% FBS was added to the lower chamber. Then cells were incubated, fixed, stained and observed, which was consistent with the migration assay as indicated above.

### Western blot analysis

Total protein was extracted after cell lysis by RIPA lysis buffer (Beyotime Biotechnology, Shanghai, China), and the protein concentration was detected using a BCA protein assay kit (Beyotime). Equal amounts of protein were added to the SDS polyacrylamide gel and transferred to a PVDF membrane (Millipore, Billerica, MA, USA). The membranes were blocked with 5% skim milk powder in TBST solution for 1 h and then incubated with primary antibodies overnight at 4 °C. Antibodies for the following were used and diluted following the manufacturer’s instructions: Bmi1 (#6964, 1:1000), AKT (#9272, 1:1000), p-AKT (phospho Ser473,#4060, 1:2000), p38 MAPK (#8690, 1:1000), p-p38 MAPK (phospho Thr180/Tyr182, #4511, 1:1000), STAT3 (#4904, 1:2000), and P-STAT3 (phospho Y705; #9145, 1:2000). The antibodies were purchased from Cell Signaling, Danvers, MA, USA, and GAPDH (AS1039, 1:1000) was purchased from Aspen, Wuhan, China. After washing three times with TBST for 10 min each time, the membranes were incubated with secondary horseradish peroxidase-coupled antibody (Aspen), visualized using an ECL kit (ThermoFisher, Waltham, MA, USA) and exposed to a gel imager. GAPDH was used as an internal control, and the gray value of each protein was calculated.

### Immunofluorescence

Both cell lines were grown in 12-well plates at a density of 8000 cells per well. After different treatments, cells were fixed with 4% paraformaldehyde for 15 min. Cells were washed three times with PBS and permeabilized with 0.25% Triton-X; then, they were blocked with 1% goat serum for 30 min at room temperature. After incubation with the primary antibodies Bmi1 (1:50) (CST) overnight at 4 °C, cells were extensively washed with PBS and incubated with the fluorescent secondary antibodies and DAPI (Beyotime). The images were visualized under fluorescence microscopy.

### SiRNA construction and cell transfection

The Bmi1 siRNA was designed and manufactured by GenePharma Co., Ltd., Shanghai, China. Double-strand Bmi1 siRNA (sense 5′-AUGAAGAGAAGAAGGGAUUTT-3′, antisense 5′-AAUCCCUUCUUCUCUUCAUTT-3′) and negative control siRNA (NC siRNA: sense 5′-UUCUCCGAACGUGUCACGUTT-3′; antisense 5′-ACGUGACACGUUCGGAGAATT-3′) were transfected into pancreatic cancer cells. SiRNA and Lipofectamine 2000 (Invitrogen, Carlsbad, CA, USA) were diluted with Opti-MEM^®^ I Reduced Serum Media (Gibco) at a final concentration of 50 nmol/L. Transfection of siRNA was performed using log phase cells when the cells were adherently grown to 30–50% in 6-well plates. The Opti-MEM medium was replaced with normal medium after incubation for 4–6 h at 37 °C and then incubated for an additional 36–48 h.

### ROS detection

According to the manufacturer’s protocol, DCFH-DA probes (Beyotime Biotechnology) were diluted in serum-free media to a final concentration of 10 μmol/L. The complete medium was replaced with the above probe-containing serum-free medium for 20 min at 37 °C, allowing the probes to enter the cells. Cells were collected then washed three times to remove the non-cell-derived probes. Then, the cells were resuspended with 200 μL of PBS and fluorescence intensity was detected by flow cytometry.

### Statistical analysis

Each experiment was repeated three times and SPSS 19.0 was used for statistical analysis. Significant differences were analyzed using Student’s t-test or one-way ANOVA, as appropriate. The results are expressed as the mean ± SD and showed statistical significance when P < 0.05.

## Data Availability

All the data is contained in the manuscript.

## Abbreviations

DAPI: 4′, 6-diamidino-2-phenylindole, dihydrochloride
FBS: fetal bovine serum
GEM: gemcitabine
HDAC: histone deacetylase
ROS: reactive oxygen species
NAC: *N*-acetyl cysteine
VPA: valproic acid
STAT3: signal transducers and activators of transcription 3
