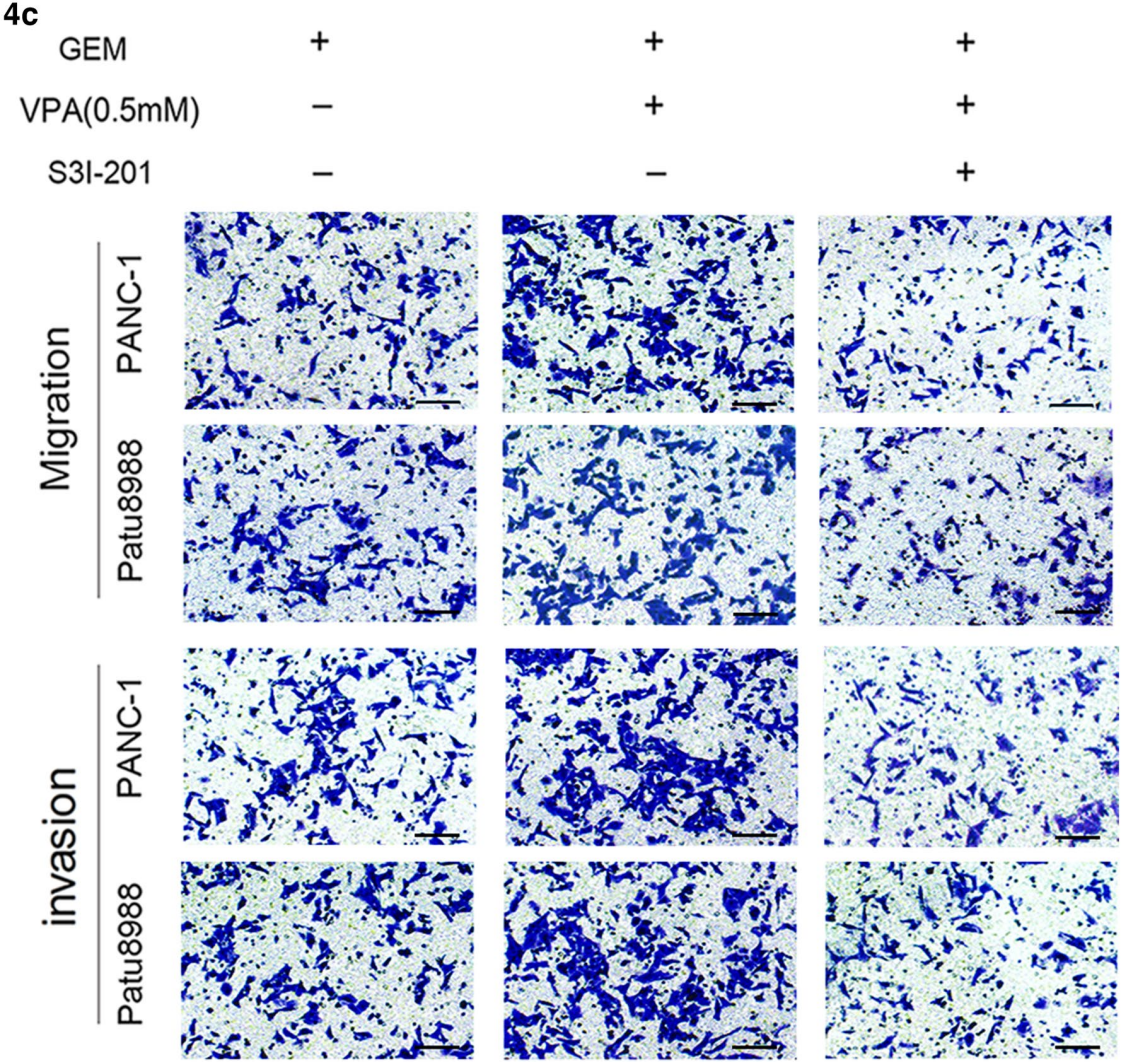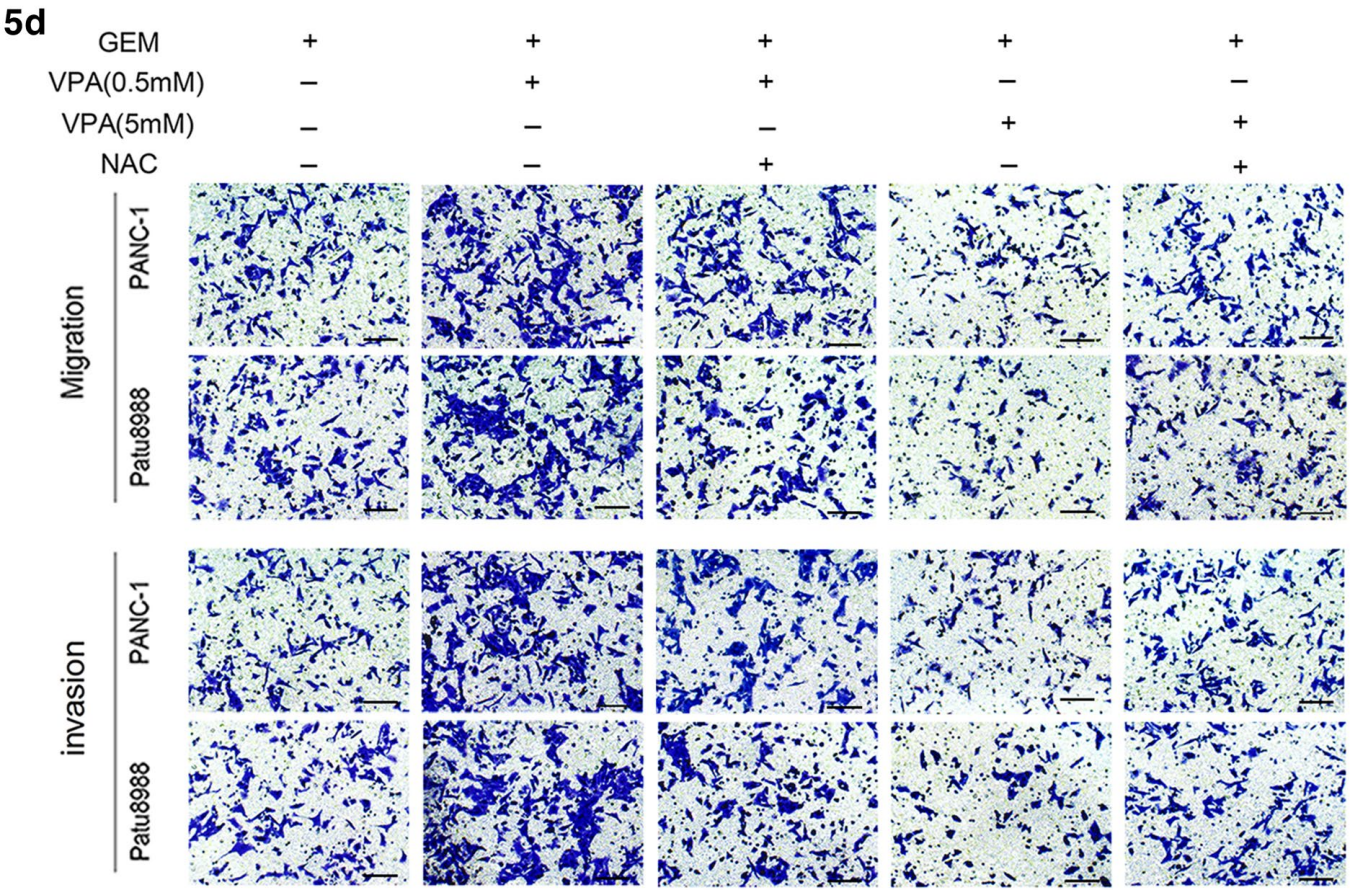# Correction: Combination chemotherapy of valproic acid (VPA) and gemcitabine regulates STAT3/Bmi1 pathway to differentially potentiate the motility of pancreatic cancer cells

**DOI:** 10.1186/s13578-023-01155-8

**Published:** 2023-11-11

**Authors:** Hehe Li, Zhengle Zhang, Chenggang Gao, Shihong Wu, Qingke Duan, Heshui Wu, Chunyou Wang, Qiang Shen, Tao Yin

**Affiliations:** 1 Department of Pancreatic Surgery, Union Hospital, Tongji Medical College, Huazhong University of Science and Technology, Wuhan, 430022 China; 2 Department of Pancreatic Surgery, Renmin Hospital, Wuhan University, Wuhan, 430060 China; 3 Department of Clinical Cancer Prevention, The University of Texas MD Anderson Cancer Center, Houston, TX 77030 USA

**Correction: Cell Biosci (2019) 9:50** 10.1186/s13578-019-0312-0

In the original publication of the article [1], the authors have noticed that a number of image files were inadvertently used or misplaced in Figs. 4 and 5 panels, likely due to processing a large number of migration or invasion transwell images in the original data folder.

Specifically, in Fig. 4c there were two images incorrectly used to represent the migration of patu8988 cells in group GEM + VPA (0.5 mM) (2nd row, 2nd column), and the invasion of PANC-1 cell in group GEM + VPA (0.5 mM) + S3I-201 (3rd row, 3rd column). In Fig. 5d, one image was used incorrectly to represent the invasion of PANC-1 cell in group GEM + VPA (0.5 mM) + NAC (3rd row, 3rd column).

The corrected figures (Fig. 4c and Fig. 5d) are given below.